# Fostering biocultural diversity in landscapes through place-based food networks: a “solution scan” of European and Japanese models

**DOI:** 10.1007/s11625-017-0455-z

**Published:** 2017-07-11

**Authors:** Tobias Plieninger, Ryo Kohsaka, Claudia Bieling, Shizuka Hashimoto, Chiho Kamiyama, Thanasis Kizos, Marianne Penker, Pia Kieninger, Brian J. Shaw, Giles Bruno Sioen, Yuki Yoshida, Osamu Saito

**Affiliations:** 1 Department of Geosciences and Natural Resource Management, University of Copenhagen, Frederiksberg C, Denmark; 2 Graduate School of Environmental Studies, Tohoku University, Sendai, Japan; 3 Chair of Societal Transition and Agriculture, University of Hohenheim, Stuttgart, Germany; 4 Department of Ecosystem Studies, The University of Tokyo, Tokyo, Japan; 5 United Nations University Institute for the Advanced Study of Sustainability, Tokyo, Japan; 6 Department of Geography, University of the Aegean, Lesvos, Greece; 7 Department of Economics and Social Sciences, University of Natural Resources and Life Sciences (BOKU), Vienna, Austria; 8 Faculty of Geosciences and Environment, University of Lausanne, Lausanne, Switzerland; 9 Graduate Program in Sustainability Science-Global Leadership Initiative, The University of Tokyo, Kashiwa, Japan

**Keywords:** Landscape stewardship, Cultural landscapes, Alternative food networks, Sustainable landscape management, Agroecology, Food systems

## Abstract

Many Japanese and European landscapes harbor biocultural diversity that has been shaped by human agency over centuries. However, these landscapes are threatened by widespread land abandonment, land-use changes, and urbanization. The aim of this study is to use a “solution scanning” method to identify place-based food networks in Europe and Japan that reinforce linkages between biological and cultural diversity in landscapes. In our analysis of 26 European and 13 Japanese cases, we find that place-based food networks are typically located in heterogeneous landscapes, are driven by civil society (and less by markets), and act at a local scale. Regional identity is the most frequently addressed societal issue. Scenery, rural tourism, and nature conservation are more important motivations in Europe, and physical well-being and revitalization of local economies are more relevant in Japan. European models are typically associated with achieving biodiversity conservation and socio-cultural tradition outcomes, and Japanese models more with public health and nutrition outcomes. We discuss the potential for transfer of approaches from Japan to Europe (e.g., models that tackle the aging of rural societies), and from Europe to Japan (e.g., models that build explicit connections between food production and biodiversity conservation). We conclude with a list of recommended policy measures, e.g., the creation of a flexible legal framework that protects the interests of and reduces political constraints for collaborative efforts to biocultural diversity in landscapes.

**Electronic supplementary material:**

The online version of this article (doi:10.1007/s11625-017-0455-z) contains supplementary material, which is available to authorized users.

## Introduction

Landscape perspectives—understanding landscape here as a spatial social–ecological system that delivers a range of ecosystem services to society (Termorshuizen and Opdam 2009)—have developed into a pivotal domain of sustainability science (Aronson 2011; Bohnet and Beilin 2015; Pearson and McAlpine 2010). Landscape is the sphere in which people and nature interact (Wu 2013), and most sustainability challenges are embedded or become visible in landscapes—for example, climate change, energy demands, health and safety, food security, urbanization, and migration (ESF 2010). Consequently, landscape perspectives are currently developing into a paradigm in global environmental and development policies (Reed et al. 2016), based on regional landscape discourses that have evolved in parallel. In Europe, these discourses have largely been framed around the European Landscape Convention (Council of Europe 2000), and in Japan mainly around the Satoyama Initiative (Takeuchi 2010).

In this study, we shed light on the commonalities of European and Japanese landscapes. European and Japanese landscapes share similar climates, and many of them are “ancient” landscapes that have not experienced major disruption by external colonization (in contrast to American or Australian landscapes) (Backéus and Emanuelsson 2016). They host similar types of farming systems, with small individual family farms being particularly important. European and Japanese landscapes comprise dynamic mosaics of settlements, arable fields, grasslands, orchards, coppice woodlands, and forests as typical land-use patterns (Hotes et al. 2015). These landscapes embody distinct features that provide humans with goods and services needed for their well-being (Garcia-Martin et al. 2017; Gu and Subramanian 2014) and include a variety of habitats and connectivity among these habitats, supporting elevated levels of biodiversity (Halada et al. 2011; Katoh et al. 2009). Biological and cultural diversity are typically closely interconnected (EEA 2010; Fukamachi et al. 2011). Despite a growing societal interest, many European and Japanese landscapes are vulnerable to economic and social changes (Hernández-Morcillo et al. 2017; Hotes et al. 2015). Driving forces such as market integration, trade liberalization, changing public policies, technological progress, aging societies, and transitions from rural to urban societies translate into tangible landscape changes, as expressed in urbanization, agricultural intensification, land abandonment, and forest expansion (Gu and Subramanian 2014; Plieninger et al. 2016).

**Fig. 1:**
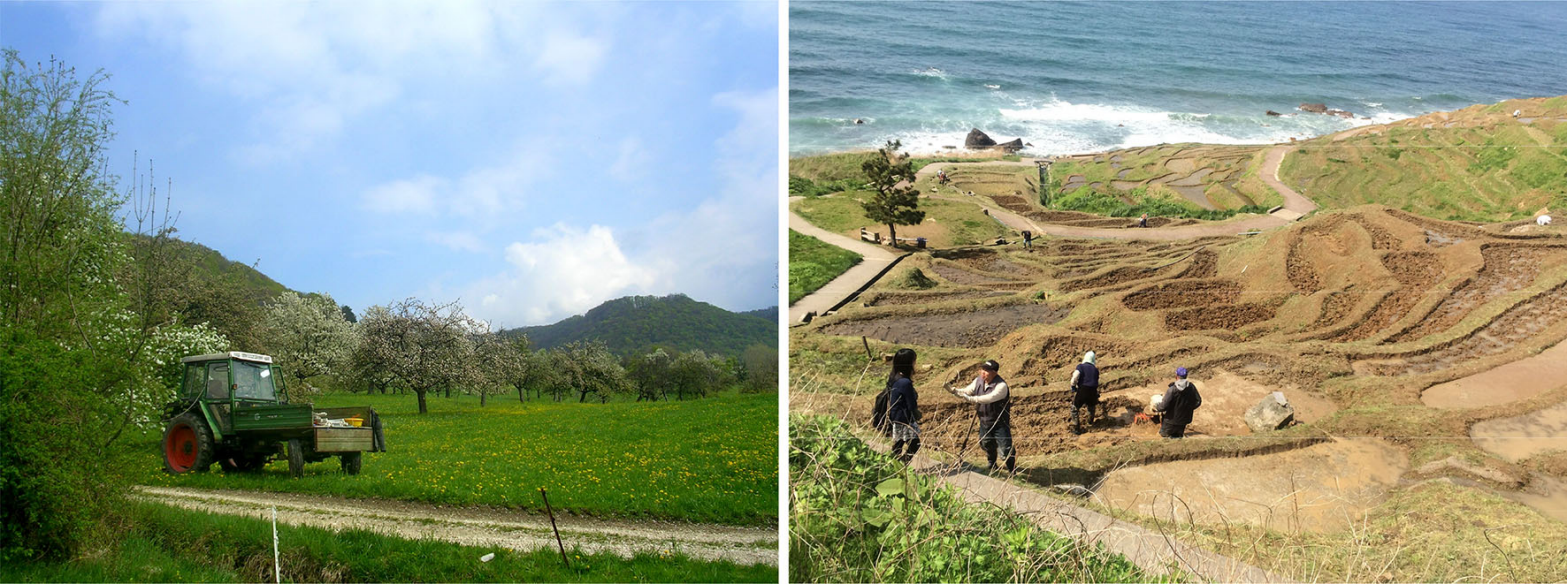
Typical place-based food networks: Reutlinger Bio-Apfelsaft initiative in Germany, contributing to the preservation of biodiversity-rich orchard meadows (c24, *left*); Tanada ownership system of rice terraces on Noto Peninsula, Japan (c27, *right*)

In Europe and Japan alike, there has been an increasing societal demand for high-quality landscapes (e.g., for landscapes that offer better opportunities for outdoor recreation and biodiversity conservation) and a general trend toward decentralized landscape planning and policy. These developments have led to the emergence of civil society-based landscape stewardship initiatives that aim for joint social, economic, and environmental objectives (Bieling and Plieninger 2017; Kuramoto 2003). Landscape stewardship is focused both on urban and rural landscapes that are exposed to multiple societal demands, and it involves multiple objectives, activities, scales, sectors, and stakeholders (García-Martín et al. 2016). Among these initiatives, the development of place-based food networks (Hedberg 2015; Holloway et al. 2006) is one of the most prominent approaches to landscape stewardship (see a European and Japanese example in Fig. 1).

The sustainability of food provision will be a key challenge for the first half of the twenty-first century, when the global food demand is expected to increase by 25–70% (Hunter et al. 2017). Three planetary boundaries—climate change, biodiversity loss, and anthropogenic nitrogen flows—have already been crossed irreversibly, all of them driven by food production (Steffen et al. 2015). The spatial concentration of farms specialized in the same products as well as the intensification of food production (with increasing nitrogen and phosphorus inputs in productive areas and land abandonment in less-favored production areas) have resulted in major land-use change and biodiversity loss (van Vliet et al. 2015). Place-based food networks are one in a series of actions that have been proposed for a more sustainable food provision, including: (a) more efficient food production and logistics systems (Schlich and Fleissner 2005); (b) organic farming, which may support the conservation of agro-biodiversity (Tuck et al. 2014); and (c) internationally implemented labels, such as globally important agricultural heritage systems (GIAHS) or social–ecological production landscapes (SEPL) which highlight the value of diverse agricultural systems adapted to different environments and a long-term commitment to nature conservation and agricultural heritage (Koohafkan 2012).

This study starts from the observation that there are striking similarities in the values that people ascribe to European and Japanese landscapes and in the challenges that these landscapes are facing. In both regions, civil society initiatives that build place-based food networks in landscapes are mushrooming, but the forms that these initiatives take show distinct differences, for example in terms of stakeholders involved, ecosystem services addressed, type of knowledge used, linkages to biophysical and cultural landscape features, understandings of human–nature relationships, and conservation mechanisms selected (Flint et al. 2013; Kieninger et al. 2011). Therefore, we argue that there is high potential for an exchange and transfer of experiences. The present study aims to perform a “solution scan” (Sutherland et al. 2014) to identify how place-based food networks in Europe and Japan create linkages between biological and cultural diversity in landscapes. Our specific objectives are:

- to collect innovative models of place-based food networks in Europe and Japan and to describe their characteristics;
- to analyze the producer–consumer relationships expressed in these networks;
- to identify the places of and flows between production and consumption in these networks;
- to analyze how place-based food networks foster biocultural diversity in landscapes.

Our paper is structured as follows. First, we review and define the concepts of place-based food networks and biocultural diversity that underlie our approach. Second, we develop a solution scanning method. Third, we present our catalog of identified food networks and analyze how these networks reinforce biocultural diversity and, fourth, we interpret our findings.

## Concepts

### Place-based food networks

Place-based or “alternative” food networks are understood as “newly emerging networks of producers, consumers, and other actors that embody alternatives to the more standardized industrial mode of food supply” (Renting et al. 2003, p. 394). This definition includes practices such as local branding, short food supply chains, farmers’ markets, local quality labeling initiatives, geographic origin labels, hobby farming, food citizenship, non-market food sharing and exchange, and education programs (c.f. Kamiyama et al. 2016; Mann and Plieninger 2017; Vandecandelaere et al. 2009; Vogl et al. 2004). The promise of these networks is that their activities translate into actual social and ecological benefits at the particular place of food production (Dennis and James 2016). Place-based food networks have developed over the recent decades as a response to the expansion of globalized agri-food supply chains that have become dominant in food markets. These developments have largely eliminated regional differences and places and distances for food products, thereby reducing direct links between people and the landscapes of food production. Our concept stresses the “place-based” character of these networks as a central property for promoting sustainability (Clark and Dickson 2003). It emphasizes their embeddedness to specific places of production, and their experiential, environmental, educational, socio-cultural, institutional, and other characteristics, which distinguish them from placeless globalized food (Follett 2009).

### Biocultural diversity

Approaches to biocultural diversity—defined as “conservation actions made in the service of sustaining the biophysical and socio-cultural components of dynamic, interacting, and interdependent social–ecological systems” (Gavin et al. 2015, p. 140)—are useful to explore linkages between food production and landscapes (Hedberg 2015). They have recently gained ground in policy and research, for example being emphasized in the “Joint Programme on the Links between Biological and Cultural Diversity” of UNESCO (Agnoletti and Santoro 2015) and in the “Charter of Rome on Natural and Cultural Capital” of the European Union (Council of the European Union 2014). Biocultural diversity involves the “diversity of life in all its manifestations—biological, cultural, and linguistic—which are interrelated (and likely co-evolved) within a complex socio-ecological system” (Buizer et al. 2016, p. 4). It is particularly helpful as a framework to assess the diverse and evolving relationships between people and nature in landscapes (Vierikko et al. 2016). For example, Gamboni et al. (2012) used such framework to explore the relations between nutrition, diversity of food products, biodiversity, and diverse landscapes in the case of the Mediterranean diet.

### Links between place-based food networks and landscapes through biocultural diversity

**Fig. 2:**
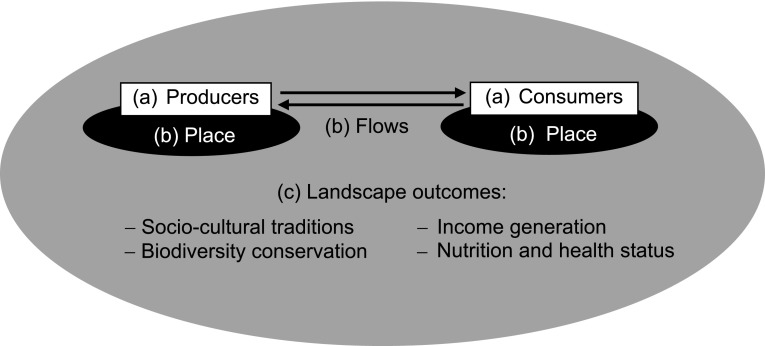
Conceptual framework comprising three dimensions of biocultural diversity: *a* producer–consumer relationships; *b* places of and flows between production and consumption; and *c* landscape outcomes

Biocultural diversity expresses the linkages between people and places (Vierikko et al. 2016). We argue that for the understanding of these complex relationships of biocultural diversity around place-based food networks and landscapes, three major dimensions have to be considered: (a) producer–consumer relationships, which define the connection between people in these networks; (b) places of and flows between production and consumption, which delineate the spatial relationships between different places where place-based food networks act; and (c) landscape outcomes that describe the multiple social–ecological impacts of place-based food networks in landscapes. We combine these dimensions as components in our conceptual framework in Fig. 2, as they offer descriptive and analytical tools per place-based food network, but at the same time accommodate comparisons over scales, spaces, purposes, and effects.

The first dimension characterizes the ways that consumers relate with food production (Vogl et al. 2004). Food producers can provide food to consumers via market relations or as a gift or non-market exchange based on reciprocity and regard (e.g., food exchange between relatives and friends) (Kamiyama et al. 2016). “Prosumers” produce parts of their diets themselves. Private or community gardens, urban “experience agriculture”, and other initiatives provide food that is usually not transferred via markets and where interaction is based on trust rather than formal quality and traceability standards and monitoring.

The second dimension captures geographical distance between production and consumption; it is focused on places, flows, and the links between them. The archetypal cases include proximity of production and consumption. They can range from “prosumption” or farm restaurants (where production and consumption take place at the same place), to local markets where producers and consumers directly interact (e.g., farmers’ markets), to cross-national or even cross-continental food supply chains where consumers typically are not able to directly interact with the producers and processors.

The third dimension illustrates the tangible outcomes of food networks in landscapes. Food is a major component of biocultural diversity, and food systems are embedded in landscapes in multiple ways, linking land management practices, biodiversity, heritage, and cultural diversity (Gu and Subramanian 2014). Principles for fostering biocultural diversity of landscapes can be derived based on insights of biocultural heritage, social–ecological systems theory, integrated conservation and development, co-management, and community-based conservation (Gavin et al. 2015). In the context of landscapes and rural development, biocultural approaches integrate multiple, frequently interlinked issues within the domains of socio-cultural traditions, biodiversity conservation, income generation, and nutrition and health status (Johns and Sthapit 2004).

## Methods

Our method, termed solution scanning, is an approach for the systematic gathering, analyzing, and prioritization of an expert-sourced list of actions specific to a problem. Such a list can be useful in a broader decision-making process to produce practical or policy interventions, or for setting research agendas (Dicks et al. 2017; Sutherland et al. 2014).

Firstly, a goal derived from normative societal concern about change or loss is defined (Pullin et al. 2013). Secondly, experts are asked to list what interventions they are aware of from their own experiences that can leverage the system toward the stated goal. Where a high variability of interventions might exist, the use of a number of experts independently supplying interventions overcomes both researcher blindness and bias. Such inclusion of experience-based interventions might be missed in a review of scientific literature alone (Fazey et al. 2006). Thirdly, the interventions are collated and redistributed to the experts, where they are assessed, cross-checked, and prioritized according to a given criteria. Solution scanning offers a fertile platform for exchange and learning for the experts involved, as it challenges their mental models on how they perceive a problem and what constitutes a solution, based on their own observations and experiences (Fazey et al. 2006).

We adapted the solution scanning method to identify place-based food networks in Europe and Japan, assessing how they foster biocultural diversity and exploring their potential for upscaling and transfer. In a first step, we defined the goal as fostering biocultural diversity in landscapes through food networks. Next, we surveyed a network of international experts in the fields of food and landscape sciences from Japan and Europe to submit, using an online form, example cases of alternative food practices from their personal or professional experiences. We chose a broad, inclusive approach to receive as wide a range of solutions as possible. In a third step, experts jointly developed the basis for the conceptual framework linking place-based food networks to biocultural diversity in landscapes at a workshop in Tokyo. Following the definition of the assessment criteria, we redistributed the cases by e-mail to the experts, who in teams of two assessed them according to the themes of interest.

We developed a list of indicators collectively to capture the three dimensions of the framework and to present, analyze, and discuss examples of place-based food networks and the biocultural diversity related to them. The list includes a wide variety of themes of interest and variables, some self-explanatory (e.g., those describing the type of product) and others that require discussion. For instance, the type of designations includes official (geographic indications) and also non-official designations, such as places, practices, producers, plants, animals, and symbols that may be used to “designate” products and food networks. Links to biocultural diversity can be applied to all variables, depending on the type of product(s) and networks.

The selection of the case studies was not always straightforward as, although all possible cases are related to a specific place, many are parts of specific registration systems (e.g., protected designation of origin, protected geographical indication, “ownership” programs, etc.), while others are related only to a specific site. We decided to treat those related to a specific registration system as one in our analysis. Another issue concerned the number of final products for each case study. Some cases refer to a single product (e.g., one geographic indication, c22), while others concerned many different products (e.g., vegetables, fruit). The typologies we used (e.g., raw or processed product) included all these individual products and therefore the number of products often exceeds those of the cases.

## Results

### Geographic context and characteristics

**Fig. 3:**
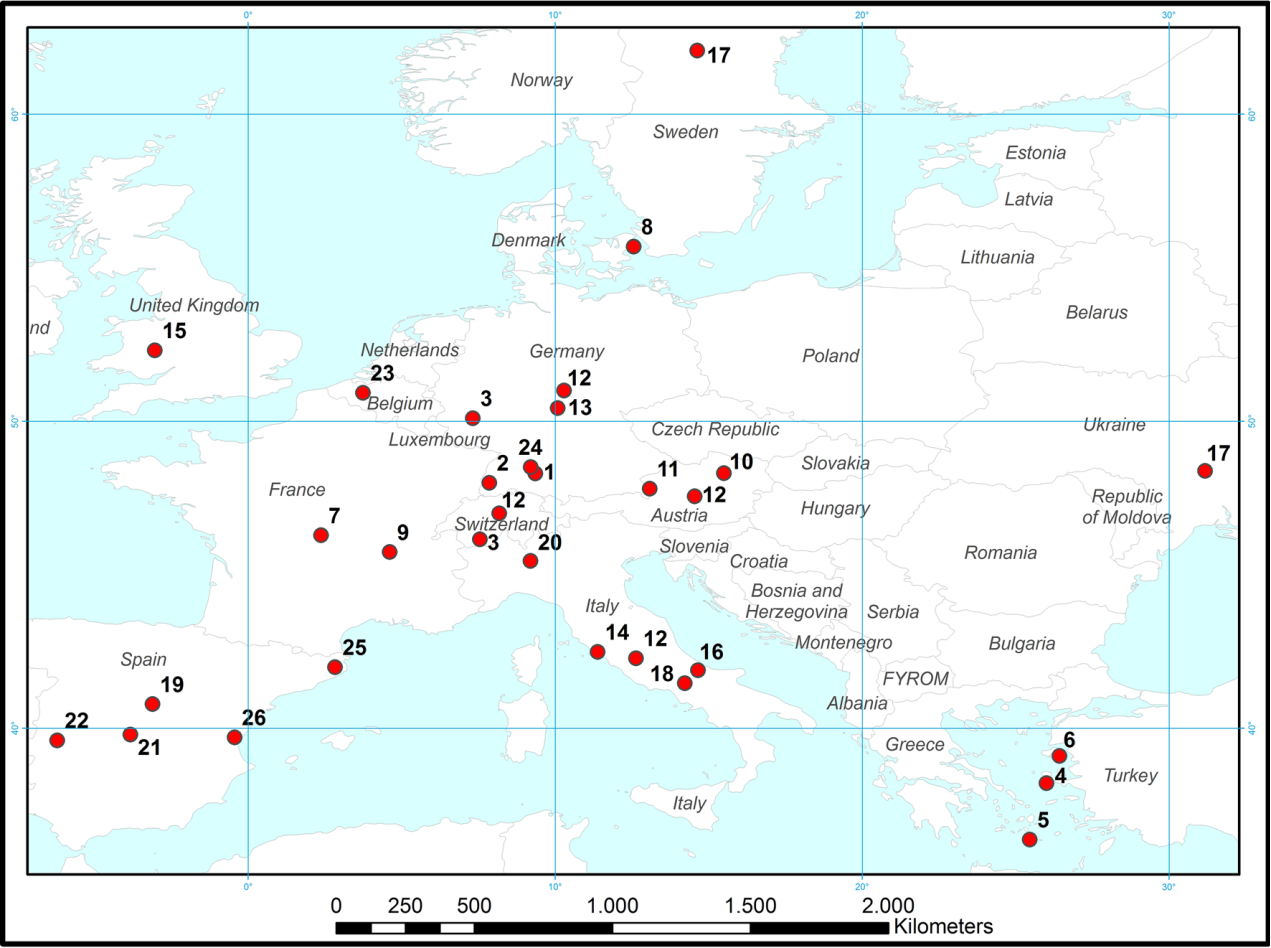
Locations of European cases

**Fig. 4:**
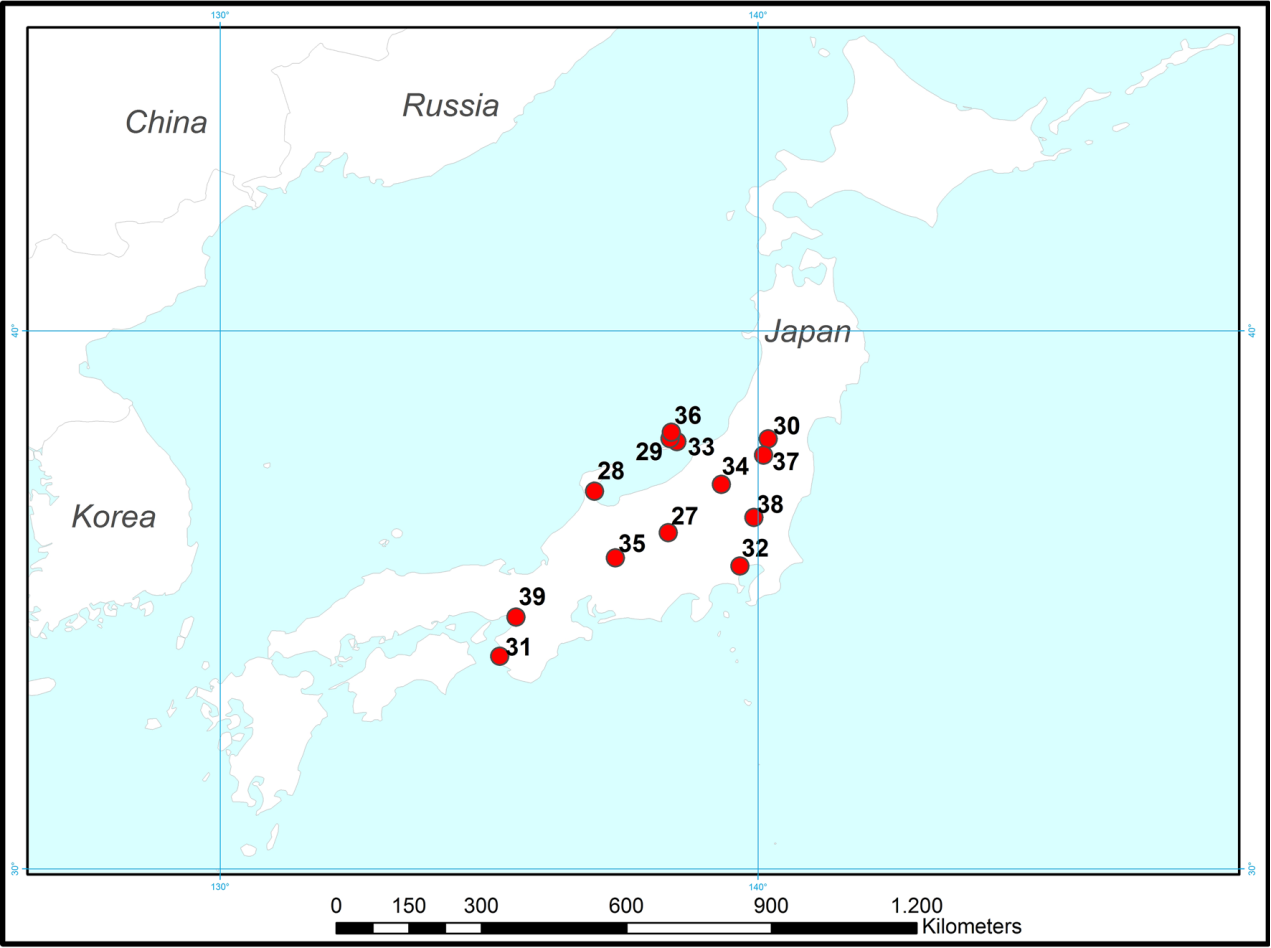
Locations of Japanese cases

Our scanning provided 39 different cases (Electronic Supplementary Material S1). The 26 European examples cover 14 countries (Fig. 3) and occur at different spatial scales, ranging from international (e.g., c12) and national schemes (e.g., c19) to regional (e.g., c14, c15) and local models (e.g., c.23). The search yielded 13 cases from Japan, covering eight prefectures (Fig. 4), mostly with a focus on farm to local scales.

Twenty-six of the models include raw products and 23 processed products. In Europe, raw products are less frequent (54% of European cases) than in Japan (92% of Japanese cases). Products are largely of terrestrial origin and include cultivated (e.g., fruits, olives, rice) and wild food (e.g., mushrooms, herbs). Marine products are completely absent in the European cases, while they account for 31% of the Japanese models (e.g., fish, oysters, algae). Forty-six percent of European cases are animal products (e.g., cheese, honey) and 73% plant products, whereas in Japan 23% cover animal and 85% plant products. Products with a long tradition (e.g., specialty ham from Spanish oak woodlands, c22) are addressed by 65% (Europe) and 62% (Japan) of the models.

Producers (sometimes organized in producer networks or cooperatives) are always included in the models, both in Europe and in Japan, while processors (e.g., creameries) are less often involved (58% of cases in Europe, 54% in Japan). Retailers and/or other businesses such as restaurants are represented similarly across the two geographical areas (42% of cases in Europe, 46% in Japan). Third parties (including certificate bodies, researchers, media, etc.) are more involved in cases in Europe (54% compared to 38% of cases in Japan). Consumers actively participate in both areas, but they do so in all Japanese cases, while in Europe they participate in 69% of the cases.

Most of the cases are strongly associated with a specific landscape, as they typically act on a local scale. Heterogeneous landscapes consisting of diverse land-use types are much more often addressed (65% of cases in Europe, 62% in Japan) than less heterogeneous (19% of cases in Europe, 15% in Japan) or uniform production landscapes (8% of cases in Europe, 8% in Japan). Heterogeneous landscapes are of great variety, covering for example Mediterranean mixed landscapes (in the case of some geographic indications such as mastic cultivation on Chios island, Greece, c4, and dehesas in Extremadura, Spain, c22), extensive pasturelands (in the case of the “rent a cow” and “cow sharing” schemes in Germany and Spain, c13, c25 and “ownership” programs in several parts of Japan, c27, c35), and coastal and marine landscapes (in the case of the *Furusato Takkyuubinn* marine food delivery activity on Sado island, Japan, c36). Uniform production landscapes mostly refer to urban agriculture cases, such as the farmer-supervised “experience garden” (c32) in Tokyo.

Governance is most typically driven by civil society (62% of European cases, 69% of Japanese cases), while markets (31% of European cases, 46% of Japanese cases) are a less prominent driver. Government-driven models are more frequent in Japan (46% of cases, compared to 23% in Europe), and, in this regard, seem to be more top-down and centrally controlled than European cases. Typically, models around small-scale local production, gardening, and “prosumers” are driven by civil society, while markets play a larger role for large-scale and less local (often processed) products. A typical example for a civil society-driven model is the widespread *Tanada* ownership system of traditional rice terraces in Japan, c27. In contrast, the commercialization of premium olive oil from Lesvos (Greece, c6) is a case for a large-scale, market-driven case.

**Fig. 5:**
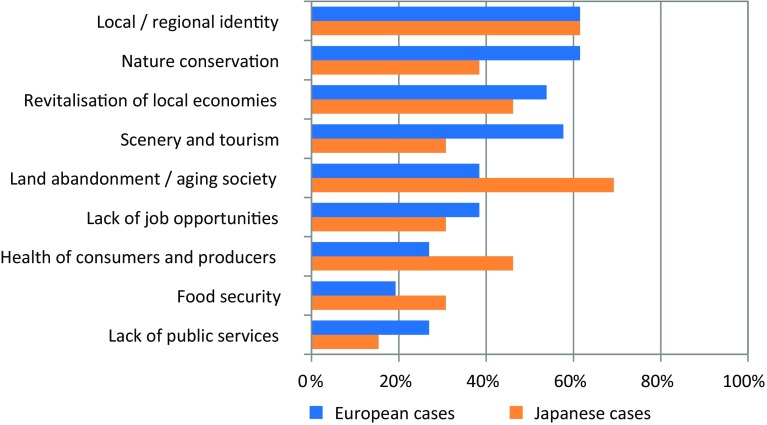
Societal challenges addressed by the cases

The cases respond to a diversity of societal issues (Fig. 5). In Europe, they most frequently address local or regional identity (62% of cases), nature conservation (62%), and maintenance of scenery and rural tourism (58%) challenges. In Japan, issues of an aging society (69%), local and regional identity (62%), physical well-being of consumers and producers (46%), and competitiveness of local production (46%) are the most important motivations behind the cases.

### Dimension 1: producer–consumer relationships

**Fig. 6:**
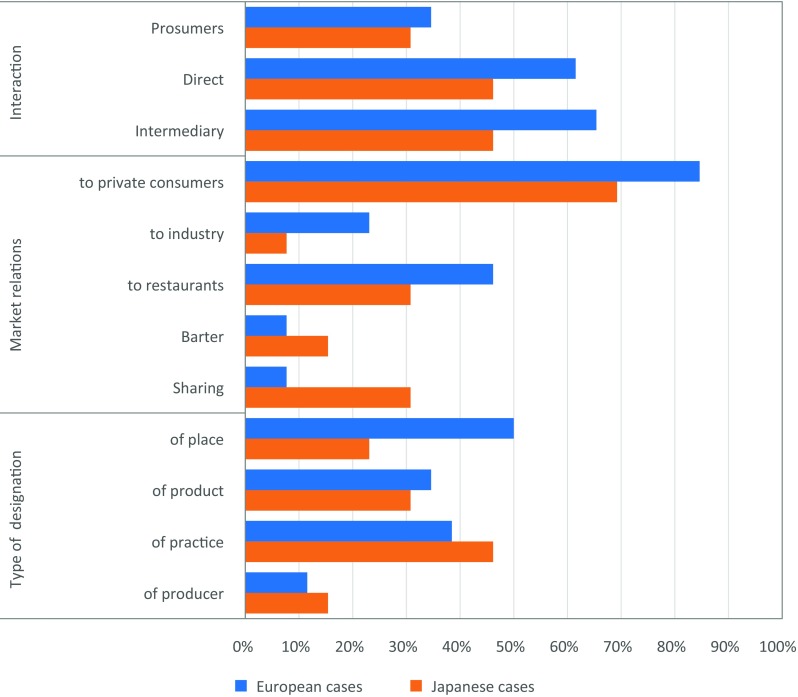
Producer–consumer relationships in the cases

Producer–consumer relationships are analyzed regarding (a) the nature of interactions between producers and consumers, (b) the market relations between them, (c) the types of designation used in these relations, and (d) the relation of the consumers with products and/or the places of production. Interaction via an intermediary has slightly more relevance (65% of European cases, 46% of Japanese cases) than direct face-to-face interaction between consumers and producers (62% of European cases, 46% of Japanese cases) (Fig. 6). Prosumption, i.e., consumption of self-produced food, plays a comparatively less important role both in the European (35%) and the Japanese cases (31%). More than half of the European cases have two or even three ways of interacting with the consumers, whereas the Japanese cases typically focus on one of the three approaches. For instance, in the “Self-experiments with a local diet” initiative (c11), a newspaper encouraged families to conduct a 6-month self-experiment, in which they would exclusively consume local food through a combination of growing, harvesting, processing, and preserving their own food (prosumption), face-to-face purchase, and purchase of food through local intermediaries (and report on this in a blog and the newspaper).

In the European cases, there is a clear dominance of market exchange—predominantly to private consumers (85%) and restaurants (46%). Only for two European cases (8% respectively), sharing and barter are observed. For the Japanese cases, market exchange to private consumers also is the most important distribution method (69%); however, we also see a comparatively high proportion of cases with barter (15%) and sharing (31%). For example, residents barter and share homegrown and foraged vegetables, fish, processed/cooked foods, gifts, and offerings for ancestral altars in the Iwakubi village case of Japan (c29). Often, these products are exchanged for labor and knowledge, and resources are frequently managed in collectives (e.g., cleaning of irrigation channels). Only 23% of the European and 8% of the Japanese models sell place-based food to the food industry.

The shares of official and unofficial/symbolic designations are higher in the Japanese cases (54% in Japanese and 42% in European cases for official designations; 31% in Japanese and 23% in European cases for unofficial designations), despite the generally lower frequency of market mechanisms there. In Europe, 50% of the cases do not have any designation, whereas this applies to only 15% of the Japanese examples. There are also differences with regard to designations: European cases most frequently (50% of cases) use designations of places (e.g., under the European Union’s protection mechanism for geographical indications, c10), whereas for the Japanese cases designations of particular production practices (46% of cases) have the highest relevance (e.g., conservation certification for Japanese crested ibis, c33, or organic certification in the “Teikei” case of Community Supported Agriculture, c30).

Both physical visits and symbolic linkages to the production area play an important role in the majority of European and Japanese cases. Whereas symbolic linkages via festivals, labels, and events are similarly important as physical visits in the European cases (77% and 81%, for example in the Mas Claperol case, c25, where people sponsor one specific dairy cow on a small farm that has a specific name, a personality, and whose products they get in exchange), the Japanese cases show a slightly lower relevance of symbolic linkages compared to visits (62% versus 69%).

### Dimension 2: places of and flows between production and consumption

**Fig. 7:**
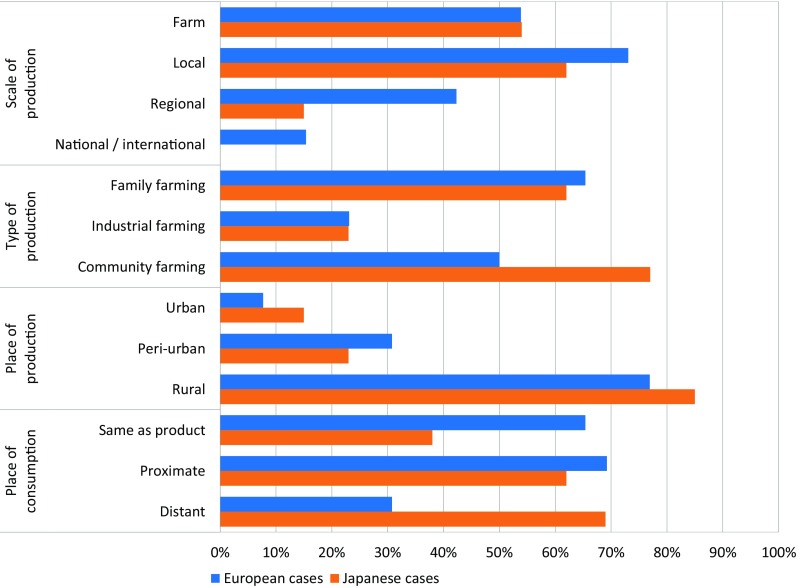
Places of and flows between production and consumption in the cases

The second dimension compares the geographical characteristics of the place-based food networks in Europe and in Japan from the perspectives of production and consumption. It analyzes (a) the scale of production (from national/international to farm scales, including coverage of multiple scales), (b) the type of production (e.g., family farming, industrial farming, community farming, full-time farming, or part-time farming), (c) the degree of rurality of place of production (ranging from urban, peri-urban, to rural areas), and (d) the place of consumption (e.g., in the area same as production, in proximity or in distance) (Fig. 7).

The local scale is the most prominent one both in European (73%) and Japanese (62%) cases, followed by the farm (54% and 54%) and regional (42% and 15%) scales. In Europe, 15% of cases act at a national or international scale, whereas no such cases were recorded in Japan. Most typically, case studies are focused on foods produced in a single scale (farm, local, regional or national/international scales) in both Europe (46%) and Japan (69%). From the cases focusing on two scales, the combination of the farm and local scales are most popular in Europe (23% of all cases) and Japan (31% of all cases). For example, the Wachauer Marille case (c10) is recognized for its apricots of protected geographical origin, covering a total of 100,000 trees. The marketing is largely organized on farm, i.e., at farm and local scales. Generally, case studies in Europe tend to involve more various and wider production scales than those in Japan.

The most important types of production that the cases are engaged in are family and community farming (65% and 50% in Europe and 62% and 77% in Japan). A typical case combining different types of production is the Oak Village restaurant in Kashiwa-no-ha, Japan, c39, which relies both on locally produced foodstuffs, but also on vegetables that patrons grow themselves and bring to the restaurant.

Rural areas are the most frequent production places of cases both in Europe (77%) and Japan (85%), followed by peri-urban and urban areas. In Europe and Japan (69% of cases respectively), most production sites are located exclusively in rural areas. For instance, wine production in Santorini, Greece, c5, is based in rural areas, but producing for middle- to high-class consumers all over the world. Here, many smallholders promote their wines under the same name based on the geographical conditions and uniqueness of to the landscape.

In Europe, most products are consumed in the same area of or in an area proximate to production (65% and 69%, respectively), followed by distant areas (31%). In Japan, proximate and distant sites are more frequent (62% and 69%, respectively), followed by the same area of production (38%). In Japan, eight cases (62%) include more than one place of consumption, while only twelve cases (46%) do so in Europe. Thus, Japanese cases tend to include more various and more distant places, while European cases tend more to the same place. An example of the former is an initiative around a local food delivery service named “Furusato Takkyuubinn” in Sado Island, Japan, c36. This initiative provides products not only to nearby consumers, but also to selected restaurants throughout Japan.

### Dimension 3: landscape outcomes

**Fig. 8:**
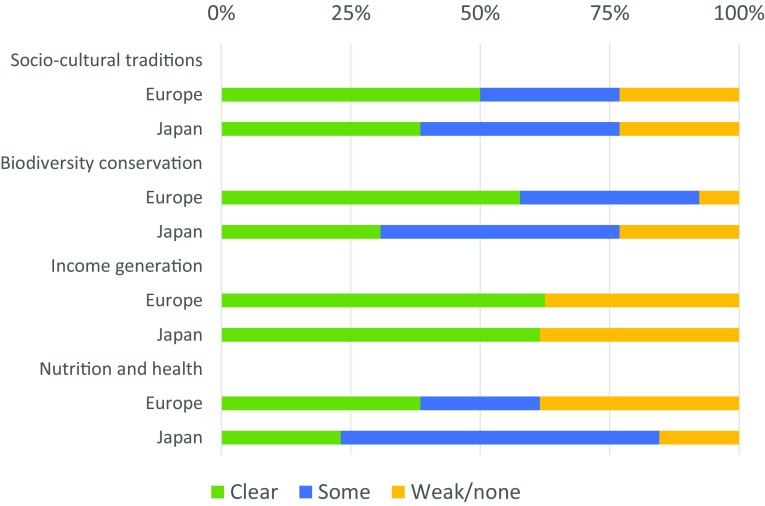
Landscape outcomes

The four aspects assessed on the contributions of the cases to biocultural diversity are (a) maintenance of socio-cultural traditions, (b) contribution to biodiversity conservation, (c) contribution to income generation, and (d) maintenance of nutrition and health status (Fig. 8).

Socio-cultural traditions are well recognized and embedded in 50% of European and 38% of Japanese cases, typically promoted through regionality, traceability, quality, and knowledge of traditional cuisines and landscape products. Many cases organize knowledge exchange, for example between generations and between rural and urban people. In the TERRAE case, c19, landowners offer their lands to unemployed people through a “Land Bank” and train them on how to become self-employed micro-farmers. In the “cow sharing” and “rent a cow” cases, c13 and c25, people are offered to visit and to take care of “their” cows. In the Rice terrace ownership system, c27, people participate in traditional rice cultivation processes, with all work carried out manually, and are taught how to produce traditional gifts, crafts, and dishes from landscape products. In the “self-experiments”, c11, people co-learn about food growing, processing, and preserving. Socio-cultural traditions are also fostered through sharing and gifting practices in non-market transactions within and beyond communities, for example through food exchange between satoyama (landscape) and satoumi (seascape), c28. Voluntary, collective landscape management work (e.g., cleaning of rice irrigation channels) and rituals (e.g., festivals and ceremonies) are also used, e.g., in c29.

The cases also show strong links to biodiversity conservation. Frequent activities include maintenance of particular seed varieties, conservation at farmland and/or at landscape scale, maintenance of habitat structures, and organic production practices. Again, linkages were more marked in Europe than in Japan, with 58% and 31% of cases reported to have a clear contribution to biodiversity conservation, respectively. Contributions to biodiversity conservation typically occur along different dimensions, namely by maintaining/fostering particular landscape features or land-use practices that are important for biodiversity, by protecting open spaces or active farmland, and by enhancing societal awareness for biodiversity conservation. For example, place-based food networks maintain key features of (agro-)biodiversity such as stonewalls and terraces (c3), scattered trees (c24), fishways (c31), or local livestock breeds (c15, c22) or foster land-use practices, such as mixed cropping systems (c1) or reduced pesticide use (c6). Some cases maintain active farmland by organizing exchange platforms for landowners (of abandoned land) and land users (interested in finding land) (c24) or by arranging land holdings in a not-for-profit trust that allows farming to occur in peri-urban areas outside the development pressures of normal market conditions (c7). Conservation awareness is promoted, for instance through linking products (apple juice, ham) to biodiversity outcomes in production landscapes sometimes supported by labels and/or certification (c3, c22, c24).

In both geographical areas, 62% of cases are assessed as contributing strongly to income generation. Most typically, income is generated through remuneration mechanisms for specific food products from landscapes. Products are frequently sold at prices higher than their mass-market equivalents, and sold through particular channels, for example through box schemes or farms shops (e.g., c7). Often, value is added through marketing processed food rather than bulk commodities (e.g., c30). Another aspect is risk sharing through pre-financing, long-term guaranteed prices or purchase guarantees, as addressed by community-supported agriculture schemes (c8, c30).

Geographical differences are most pronounced in how European and Japanese cases relate to people’s nutrition and health (with 62% of European, 85% of Japanese cases showing a clear or some contribution). In the Grand Parc Miribel Jonage, c9, recreation possibilities for local people have been enhanced by improving access to nature and culture in landscapes for residents of the Lyon metropolis. In rural communities of Japan, non-market food exchange is inherent in the traditional lifestyle. In addition to diversifying community members’ nutritional intake in general, these informal networks offer a safety net to elderly people to sustain their nutrition, access to food, and health (c28, c29). While most case studies diversified and enhanced consumers’ nutritional intake by providing access to varied agri-food supply chains, health-based motives were more evident in the Japanese case studies surveyed (e.g., c30).

## Discussion

### Place-based food networks in Europe and Japan

This study responds to calls from science and policy to identify, replicate, and upscale integrated approaches that foster biocultural diversity of landscapes. We particularly focus on place-based food networks as an emerging approach to valorize distinct landscape characteristics. We selected 26 European and 13 Japanese cases that we are familiar with and that we believe are representative of the diversity of approaches. Our European and Japanese cases share some fundamental similarities. For example, cases are most typically located in heterogeneous landscapes, are driven by civil society (and less by markets), and act at a local scale. By that, they contrast prevailing tendencies in current food systems toward long-distance value chains, with transnational corporations playing an important role (Sundkvist et al. 2005). Consumers expect short supply chains to be more sustainable due to a number of reasons, such as (a) trust and transparency supported by face-to-face interaction; (b) consumers’ insights into and sometimes even opportunities to co-define local production standards; (c) the conservation and development of place-based skills, recipes, breeds, varieties, and diets; and (d) associated benefits for landscape diversity and identity building (Campbell 2009; Hinrichs 2003; Sonnino 2013). However, these and other sustainability benefits of place-based food networks often show trade-offs with aspects of efficiency, as recently quantified for Mediterranean farming systems (Rodríguez-Ortega et al. 2017). In particular, the climate effects of shorter food miles have been controversially discussed regarding trade-offs between less transport-related emissions on the one hand and the energy-inefficiency of decentralised production and logistics systems on the other (Schlich and Fleissner 2005).

In our cases both in Europe and Japan, regional identity is the most frequently addressed societal issue. However, we also find strong cultural differences. For example, raw products and marine food are much more prominent among Japanese cases, while processed, terrestrially produced food dominates the European cases. Non-market exchange (e.g., through bartering or sharing) of food, but also the use of designations, is more widespread in Japanese cases. We also found clear differences in motivations, with scenery, rural tourism, and nature conservation being more important for European cases, and physical well-being and revitalization of local economies being more relevant for Japanese cases. European cases seem to be more related to achieving biodiversity conservation and socio-cultural tradition outcomes, and Japanese cases more to public health and nutrition outcomes.

**Fig. 9:**
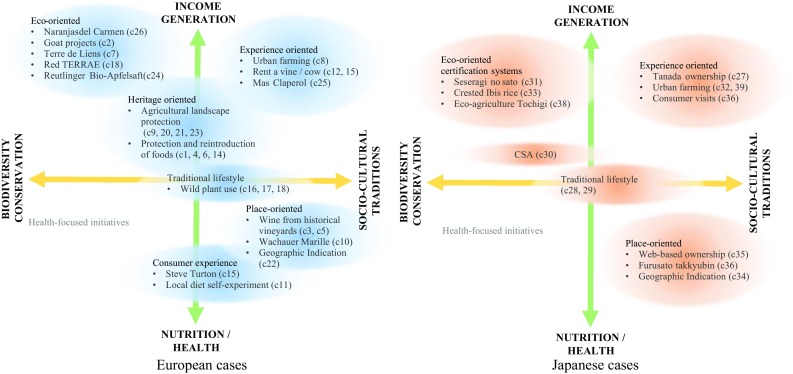
Illustrative clustering of European and Japanese cases

Following the biocultural framework by Johns and Sthapit (2004), our analysis loosely classified cases by whether their main focus is on the production (income generation) or consumption (nutrition/health) side, and whether their conservation emphasis and impact are more on biodiversity or on socio-cultural traditions. Overall, case studies in Europe and Japan show similar distributions across the quadrants (Fig. 9). In Europe, the continuum of cases with foci on the biodiversity impacts of production processes, conservation of landscape and food heritages, and cases emphasizing the cultural implications of place-based products are more pronounced. Certification systems stand out in Japan, and were broadly categorized for their emphasis on ecologically oriented practices or on the conservation of traditional products and landscapes. In both regions, initiatives based on embedded consumer experiences in the production landscape were placed in the quadrant of production and culture. No cases were placed in the quadrant of biodiversity and consumption, as initiatives with a primary focus on consumer health were outside our focus on place-based food networks. Finally, as discussed above, the biocultural aspects of traditional place-based food networks were most integrated, so that these cases were closest to the equilibrium of orientations analyzed here.

### How do place-based food networks relate to landscapes through biocultural diversity?

Our cases suggest that landscape characteristics are both outcomes and drivers of place-based food networks. Less-productive mountain areas, for example, face difficulties in competing on price-driven commodity markets. For farmers located there, place-based food networks provide alternative marketing channels that potentially link them to consumers who are willing to pay more for immaterial product qualities, such as the conservation of biocultural diversity. Therefore, landscape characteristics may drive the development of the place-based food networks analyzed on the one hand. On the other hand, place-based food networks can be influential in shaping landscapes (Ilbery and Kneafsey 1999; Ilbery et al. 2000; Marsden et al. 2000). These landscape impacts are not “by-products” or unintended outcomes of place-based food networks. Rather, the networks deliberately practice landscape stewardship, as defined by Bieling and Plieninger (2017). In particular, place-based food networks are able to act as critical links between different influencing factors of food and landscape quality, farming, forestry, ecosystem management, and socioeconomic change. Place-based food networks can also be an important nexus for creating connections of people to landscapes within and across scales (c.f. Sundkvist et al. 2005).

### Limitations of our approach

Our solution scanning approach involves a diversity of landscape and food systems researchers from a variety of countries. The approach is exploratory in nature, and the solution scanning method generally has low repeatability (with possibly a different set of cases being covered if the exercise would be repeated) and a moderate risk of bias (Dicks et al. 2017). In particular, there is the possibility of a bias toward certain geographic regions and cases. For example, countries such as Austria and Greece—where some of the researchers are based—are strongly represented, whereas regions such as Eastern Europe are less so. Also, our cases may be biased to the local scale. However, we tried to minimize the risk of bias by including a broad set of experts with strong and diverse experience in place-based food networks. Further, we did not address issues of cost-effectiveness and potential trade-offs such as those between suppliers and consumers, and between food provision and different ecosystem services. For a clearer picture of the multiple landscape outcomes of place-based food networks, a more comprehensive inventory of traditional and innovative models needs to be carried out.

### Potential for transfer between Europe and Japan and for upscaling

Despite many similarities and some context-specific particularities (e.g., the prevalence of marine products that is rooted in Japanese fish-eating cultures), our results point to some specific Japanese models that might be transferred to Europe, and to some European models with potential for application in Japan. Ideas that might be brought to Europe include models that tackle the aging of rural societies and rural depopulation at large, for example by involving urban residents in the management of rural landscapes (c27). Japan may also provide role models for making more comprehensive use of innovative certification systems (c33), for non-market exchange systems (c28), and for combining food production with sustainability-oriented education (c32). From the European cases, there may be potential for transfer to Japan related to models that successfully integrate scenery and tourism (c2), models that build explicit connections between food production and biodiversity conservation (c24), and models that link to cultural heritage (c3, c23). The ability to scale up cases clearly depends on the type of food networks. Although generalizations are not always feasible, cases in the upper half quadrants (income generation side of Fig. 9) tend to present limitations to scaling up, since they are closely associated with specific production systems and/or localities. In contrast, cases in the lower half quadrants (nutrition/health side of Fig. 9) can scale up beyond the boundary of the production system as long as consumption demand levels do not exceed the levels of sustainable production. The effects or interactions in this realm of nutrition/health at different scales are areas under development and subjects of future research.

### The way forward

In the study, we present a number of cases where place-based food networks and landscapes are closely connected, using biocultural diversity as our analytical lens. Place-based food networks relate to landscapes through biocultural diversity approaches that foster biodiversity conservation, socio-cultural traditions, income generation, and nutrition and health status of people. The precise contribution of place-based food networks to biocultural diversity varies substantially from case to case, according to agro-ecological conditions, resource scarcity, resource ownership regime, characteristics of the food value chains, and other factors (le Polain de Waroux and Lambin 2013). Rather than establishing quantitative evidence for these contributions, our study intends to display the variety of different models of place-based food networks in landscapes of different contexts.

Although the state was not an important driver of most cases and the public funding that they receive is typically small, many initiatives depend crucially on this modest public policy support. Further replication and upscaling require a sound institutional framework. Agricultural policy schemes, regulations on geographic indications and other certificates, tax exemptions, or direct financial subsidies can be part of such a supportive public policy framework. Taking up experiences from integrated landscape management in Europe (García-Martín et al. 2016) and accepting that place-based food networks fulfill important public interests and cultural identities; such support from science and policy might be done through the following pathways:

- further evidence-based scientific assessment of place-based food networks and their multiple contributions to fostering biocultural diversity in landscapes as well as potential trade-offs through systems approaches;
- strengthening of efforts to raise societal awareness of existing models and to enhance the capacity for fostering biocultural diversity in landscapes;
- creation of a flexible legal framework based on the knowledge and experiences generated by place-based food networks, to protect the interests and reduce political constraints for collaborative efforts to biocultural diversity in landscapes;
- definition of local quality standards complementing the abundance of (inter-)national food standards, to ensure the diversity of locally adapted breeds, varieties, cultivation, and processing practices;
- advancement of existing labeling and certification approaches to reinforce linkages between quality products, distinct production processes, and biocultural diversity in landscapes also beyond local scales, engaging consumers for landscape stewardship across larger geographic distances; and
- stronger consideration of place-based food networks in international trade and discourses on global food security.

## Electronic supplementary material

Below is the link to the electronic supplementary material.

Supplementary material 1 (DOCX 20 kb)
