# Supplementary material for: Fostering biocultural diversity in landscapes through place-based food networks: a “solution scan” of European and Japanese models
Source: Sustain Sci. 2017 Jul 11;13(1):219–33. doi: 10.1007/s11625-017-0455-z (PMC6086265; doi:10.1007/s11625-017-0455-z)
Supplement: Supplementary file 1 — Supplementary material 1 (DOCX 20 kb) [file 11625_2017_455_MOESM1_ESM.docx]

**Electronic Supplementary Material S1:**

**Description of the names, principles, and countries of the cases**

| **No.** | **Case name** | **Case principle** | **Country** |
| --- | --- | --- | --- |
| 1 | Alb Leisa | Rediscovered local lentil variety, cultivated in a mixed organic cropping system. Strong connections to cultural heritage, agrobiodiversity and local identity. | Germany |
| 2 | Goat projects | Goat pasturing on marginal, often biodiversity-rich and/or culturally relevant grasslands, in order to prevent reforestation. Often with involvement of volunteers from the local community. | Germany |
| 3 | Wine from historical terraced vineyards | Viticulture on terraced vineyards with interspersed drystone walls. Practices like manual harvest and low or no herbicide inputs contribute to a rich biodiversity and a high wine quality and sustain the cultural heritage. | Germany, Switzerland |
| 4 | Mastic cultivation | Mastic cultivation (resin of *Pistacia lentiscus*) that is used to produce a wide variety of products and medicine and has managed to make its cultivation again profitable. | Greece |
| 5 | Wine production | Wine produced with regional variety of vines and sold to tourists - exported as high quality. Has kept vine cultivation on the island active. | Greece |
| 6 | Olive oil production | Olive oil produced by local variety of olive trees with very extensive practices that contributes significantly to biodiversity and ecosystem services, but faces abandonment. | Greece |
| 7 | Terre de Liens | Land-trust which buys agricultural land and rents long-term to farmers with conditions that they manage for biocultural sustainability. | France |
| 8 | Østergro Urban Farm | Community Supported Agriculture scheme with rooftop farm within the city and production farm outside of the city. Aim is to explicitly connect urbanistes with the source of their food and provide a sustainable food centred community centre in the city | Denmark |
| 9 | Grand Parc Miribel Jonage | Multifunctional peri-urban park. Because it holds drinking water for the city of Lyon, agricultural production in the park happens without pesticides, herbicides, and artificial fertilizers. Products are sold under a park label at farm shops within the park as well as being distributed in the city of Lyon. | France |
| 10 | Wachauer Marille | The terraced world heritage landscape Wachau has been shaped by centuries of wine and apricots production. The apricot is marketed with protected geographical indications, based on locally defined quality standards and product specifications. | Austria |
| 11 | Self-experiments with local diet | Seven fFamilies in Salzburg experimented with a local diet. They exclusively sourced food from nearby farmers, processed and stored their own food and thus experienced the link between landscape and the seasonal availability of food. | Austria |
| 12 | Rent a vine | Volunteers (oenophiles) rent some grape vines and participate, under instruction of the vinter, in all steps of winemaking, in order to produce their own wine. | Austria, Germany, Italy, Switzerland |
| 13 | Cow sharing | Citizens (via the non-profit organisation “Osnabrücker Weidelandschaften”) hold on an extensive and ecological worthwhile pastureland of the city Osnabrück some cows and share their meet at the end of the season. | Germany |
| 14 | Saffron re-introduction | Re-introducing and branding of locally produced saffron which was recently discovered to have been cultivated in ancient times in south Tuscany, Italy. | Italy |
| 15 | Steve Turton meats network | Locally produced sausages in good quality in a tracable system, developed in the context of the occurrence of the BSE disease. The biosecurity crisis raised the need to emphasize the peculiar qualities of locally-grown stocks and products in the South West of England. | United Kingdom |
| 16 | Wild plant use | Traditional food and herbal uses of wild plants in the ancient South-Slavic diaspora of Mundimitar/ Montemitro. | Italy |
| 17 | Wild food and medicine harvesting | Utilization of non-timber forest products for wild food and medicine in rural landscapes. | Sweden, Ukraine, Russia |
| 18 | Wild food consumption | Recording of local knowledge concerning traditional uses of wild food plants and related practices, such as gathering, processing, cooking, therapeutic uses, with the aim of preserving an important part of the local cultural heritage. | Italy |
| 19 | Red TERRAE | Counteracts land abandonment through fostering cooperation among local administrations, landowners, farmers, experts, and citizens, thereby enhancing agricultural biodiversity and self employment. | Spain |
| 20 | Parco Agricolo Sud Milano | Coherent management of the agricultural land in Milano’s metropolitan area bridging nature conservation, cultural traditions, agriculture, rural tourism and recreation to improve locals livelihoods, fostering localised food production and consumption, and protecting the environment. | Italy |
| 21 | Asociación Trashumancia y Naturaleza | Maintaining and revitalizing traditional use of livestock drove roads by fostering the economic viability of transhumance to support their social, cultural and ecological functions. | Spain |
| 22 | Dehesa de Extremadura | Ham produced by regional pig breed and that is based on feeding pigs with acorns. Incentivices farmers to keep oaks on their lands. | Spain |
| 23 | Ijzer & Polder | Cultural landscape title enforced for fostering identity, product promotion, and targeted policies. | Belgium |
| 24 | Reutlinger Bio-Apfelsaft | Locally produced apple juice contributing to preservation of biodiversity-rich orchard meadows. | Germany |
| 25 | Mas Claperol | Commercialisation and crowdfunding of dairy products relying on the personal involvement of each consumer to bridge animal rights, environment protection and economic viability. | Spain |
| 26 | Naranjas del Carmen | Applying innovative production techniques and innovative marketing channels based on crowd-farming to make the production of ecologic fruits profitable. | Spain |
| 27 | Tanada Ownership System | Ownership system of rice terraces, where non-residents rent and maintain a field on a yearly basis. | Japan |
| 28 | Homegardens and communal sharing | Home food production and informal sharing of food without market transaction within and beyond communities. | Japan |
| 29 | Neighborly food exchange | Informal sharing of home-grown and cooked foods within a traditional rural community. | Japan |
| 30 | Teikei | Community supported organic agriculture initiated by a group of young rural farmers and urban mothers. | Japan |
| 31 | Seseragi-no-sato  (or “fish-cradle paddy rice”) | Rice is produced in paddy field which serves as fish spawn field by reduced use of agrochemicals and ensuring fish movement between paddy and outer water systems, the linkage once was cut under modernization of agriculture. | Japan |
| 32 | Taiken-nouen | Rational farmland conservation scheme in urban areas by a collaboration of farmer and non-farmer resident. | Japan |
| 33 | Japanese crested ibis conservation certification | Certification of ecologically produced rice for the conservation of endangered Japanese crested ibis. | Japan |
| 34 | Dried persimmon production | Registration of dried persimmon under the Geographical Indication(GI) protection system with the production process and quality control registered at the national level. | Japan |
| 35 | Food ownership system | Food ownership systems aggregated as in a catalogue for urban consumers. | Japan |
| 36 | Furusato Takkyuubinn | Food delivery system managed by general incorporated association based in local area for encouraging local farmers and fishermen. | Japan |
| 37 | Experiential rice farming | Rice farming as ecological tourism, primarily for the education of urban children. | Japan |
| 38 | Eco-agriculture Tochigi | Both consumers and producers declare to promote environmentally friendly agriculture | Japan |
| 39 | Oak Village | Family and community farming for neighboring residents that provides locally grown products for the restaurant. | Japan |
